# New insight into CCN3 interactions - Nuclear CCN3 : fact or fantasy?

**DOI:** 10.1186/1478-811X-4-6

**Published:** 2006-08-08

**Authors:** Bernard Perbal

**Affiliations:** 1 Laboratoire d'Oncologie Virale et Moléculaire, UFR de Biochimie, Université Paris 7-D.Diderot, 75005, Paris, France

## Abstract

The identification of potential partners for CCN3(NOV) sheds new light on the biological activity of this signaling protein. In particular, the physical interaction of CCN3 with the IL33 cytokine combined with previous data indicating that CCN3 expression was regulated by TNFalpha and IL1 cytokines, point to CCN3 as a potent player in a variety of inflammatory responses, including neurodegenerative disease, and arthritis. Nuclear proteins that are involved in the regulation of RNA processing and chromatin remodeling were also found to interact with CCN3. These observations reinforce the concept that routing of CCN3 to the cell nucleus where it acts as a transcription regulator, might constitute a key element in the balance between the anti- and pro-proliferative activities of CCN3 proteins.

## 



## Background

Cellular biological processes are governed by several interconnected signaling pathways that allow fine tuning of proliferation, growth arrest, differentiation, and death. Central to this integrated control is a hierachy of regulatory circuitry that likely requires the interplay of multi-protein complexes. Multimolecular complexes are known to provide flexibility and subtle integrated responses to a variety of paracrine or autocrine signals that may be synergistic and antagonist.

In the past decade, a new family of signaling proteins, known as CCN proteins, has emerged [1,2]. These secreted matricellular proteins are believed to play key roles in several fundamental biological processes, among which cell proliferation, differentiation and apoptosis have been the most studied [3]. Their involvement in osteogenesis, angiogenesis, regeneration, fibrosis and cancer development is now well documented, and we have previously proposed [1,4] that the CCN proteins might act as scaffolding or adaptor proteins that would permit the interconnection between independent signaling pathways that is required for balanced biological processes.

In spite of the increasing amount of interest devoted to CCN proteins, very little is known regarding their mode of action. This is probably due to the fascinating multimodular organisation of the CCN proteins with a high cystein content and a well-known tendency to aggregate, that make it difficult to obtain large amounts of pure protein and to develop reliable reagents. Reports have flourished demonstrating detection of CCN RNAs and proteins in various organs, increased or decreased expression of CCN proteins in normal and pathological conditions, association of CCN expression with differentiation or proliferative status in given tissues, but very few have addressed key questions such as to the precise functions of CCN proteins, their mechanisms of action, and whether they are genuine signaling molecules or co-factors.

Very elegant work published by the groups of L. Lau and K. Lyons [5,6] has identified defects resulting from knock out of CCN1 and CCN2, thereby shedding light on the critical functions of these two proteins in osteogenesis and angiogenesis. Despite the considerable progress that was brought by these reports the mode of action of CCN1 and CCN2 remains elusive. Several publications have reported the identification of proteins that physically interact with CCN proteins [3]. The use of full length recombinant proteins or variants lacking specific structural domains did not permit, as yet, to assign biochemical function(s) common to the CCN proteins.

In an attempt to identify partners of the CCN proteins that might provide clues in the search for CCN proteins biochemical functions, we had previously used the yeast 2 hybrid strategy [7,8]. The results that were obtained suggested that CCN3 interacted with several different types of proteins and raised the possibility that in addition to partners localized at the cell membrane or in the extracellular matrix, the CCN3 protein might interact with the transcription machinery in the nucleus of cells.

We now report on the analysis of an additional set of clones that were positive in the two hybrid screening that identify potential CCN3 partners suggesting other signaling pathways in which CCN3 might be acting. This data highlights the wide range of interactions in which CCN3 is involved, and reinforces the idea that nuclear CCN3 is a putative regulator of transcription.

## Methods

### Two hybrid system screening

Conditions for screening the two hybrid system libraries have been described previously [7,8]. Positive clones were restreaked twice on selective medium deprived of histidine and containing 25 mM 3-amino-1,2,3,4 triazole (3AT), before checking interactions with the β-galactosidase assay. Recombinant plasmids encoding the proteins interacting with CCN3 were purified from 4 ml of yeast grown overnight in minimal medium. Cells were centrifuged in microcentrifuge tubes and resuspended in 100 μl of lysis buffer (2% Triton X100, 1% sodium dodecyl sulfate (SDS), 100 mM NaCl, 10 mM Tris/HCl, pH 8.0, 1 mM EDTA, pH 7.5), 100 μl of sterile glass beads, and 100 μl of a phenol/chloroform/isoamyl alcohol mixture (49.5/49.5/1). After two minutes of vortexing, the mixture was centrifuged for 20 minutes at 25°C and the upper phase used as a source of DNA.

A series of clones that had not been analyzed in the previous studies, were expanded, and each individual clone was streaked three consecutive times on plates containing selective medium. DNA purified from each positive clone was used to transform E. Coli HB101. Transformed bacteria auxotrophic for the leucine marker and resistant to ampicillin, were expected to contain plasmid encoding a potential partner of CCN3. The plasmid DNAs were purified on Quiaquick columns. DNA sequencing was performed by Genomex (Grenoble) and the sequences were analyzed with the BLAST program (National Center for Biology Information).

### Transformation

Electrocompetent cells were prepared by seeding one litre of LB medium with 10 ml of an overnight culture of the appropriate bacterial strain. The cells were grown with shaking at 250 rpm at 37°C until the culture reached an optical density of 0.7 and they were then collected by centrifugation at 4000 *g *in a prechilled GS3 Sorvall rotor for 15 minutes. The bacterial pellet was resuspended in one litre of ice cold sterile 10% glycerol and the suspension was centrifuged at 4000 *g *in the GS3 Sorvall rotor for 15 minutes at 4°C. The pellet was then resuspended in 0.5 litre of ice cold sterile 10% glycerol and centrifuged again at 4000 *g *for 15 minutes at 4°C. The pellet was resuspended in 20 ml of ice cold sterile 10% glycerol, and centrifuged again. The final pellet was resuspended in 2 ml of ice cold sterile 10% glycerol and the resulting preparation of electrocompetent cells was distributed in 100 μl aliquots and frozen at -80°C. The competence of the bacterial preparations was checked by performing a transformation and counting the colonies obtained with a known amount of pUC18 DNA. The ligation of vectors and inserts to be cloned was usually performed for 18 hours at 12°C in 50 μl reaction samples containing 10 nM ends of each DNA fragment and 1 μl of a high concentration T4 DNA ligase (New England Biolabs, Beverley, Massachusetts, USA; 400 U/μl). For transformation, ligation mixtures were mixed with 0.5 ml of electrocompetent cells in electroporation cuvettes (Biorad, Marnes La Coquette, France) on ice. Electroporation was performed with Biorad Gene pulser (capacitance, 25 μF; resistance, 300 Ohms; at 1.8 KV). Electroporated bacteria were resuspended in 1 ml of chilled LB medium, incubated for one hour with agitation (250 rpm) at 37°C, and spread on to 12% agar plates containing 100 μg/ml ampicillin.

### Bacterial strains

For the expression of the GST fusion proteins, BL21 bacteria (*Escherichia coli *BF- ompT hsdS(rB- mB-) dcm+ Tetr gal (DE3) endA Hte) were used. HB101 bacteria (F- supE44 ara14 galK2 lacY1 leuB6 thi-1 (gpt-proA)62 rpsL20 (Strr) xyl-5 mtl-1 recA13 (mcrC-mrr) HsdS 20 (r- m-)) were used to recover, from transformed yeast, the plasmid DNA encoding the GAL4TA domain fused to the target protein selected in the two hybrid screening. The cloning and amplification of the other plasmids were performed in DH5 (F'phi80dlacZ delta(lacZYA-argF)U169 deoR recA1 endA1 hsdR17 (rk-, m k+) phoA supE44 lambda-thi-1 gyrA96 relA1/F' proAB+ lacIqZdeltaM15 Tn10(tetr))

### DNA preparation

The preparation of recombinant DNA was performed with Qiagen kits. After performing the optional washing of the columns, the recombinant plasmid DNA from the minipreparations was usually eluted with 50 μl of sterile distilled water. For midipreparations, the final DNA pellet was resuspended in 300 μl of sterile distilled water.

### Production and purification of GST fusion proteins

A 20 ml sample of an overnight culture of recombinant bacteria grown in LB culture medium containing 100 μg/ml ampicillin was used to seed one litre of ampicillin LB medium. The culture was incubated at 37°C with agitation at 250 rpm until an optical density of 0.6 was reached. At that time, IPTG was added to a final concentration of 0.1 mM to induce the production of the GST fusion protein, for 90 minutes at 37°C with agitation. Bacteria were collected after 10 minutes of centrifugation at 4000 × *g *in a Sorvall GSA rotor at 4°C and resuspended in 50 ml lysis buffer (10 mM Tris/HCl, pH 7.5, 100 mM KCl, 1 mM EDTA, 5 mM dithiothreitol (DTT), and 0.5% NP40), containing 100 mM phenylmethylsulfonyl fluoride (PMSF), 10 mM TPCK (*N*-tosyl-L-phenylalanine chloromethyl ketone), and 10 mM TLCK (*N*-*p*-tosyl-L-lysine chloromethyl ketone). After adding 5 ml of lysozyme solution (10 mg/ml in 25 mM Tris/HCl, pH 8.0) the mixture was sonicated until the cells were completely broken and centrifuged for 20 minutes at 5800 × *g *in a Sorvall SS34 rotor to eliminate the cell debris. Each of the two 25 ml fractions of supernatant was mixed with 1.5 ml of GST sepharose beads (50% slurry in phosphate buffered saline (PBS)), and incubated for one hour at 4°C on a rotating wheel. The GST beads were centrifuged at low speed in a table top centrifuge and washed five times with 6 ml binding buffer (20 mM Tris/HCl, pH 7.5, 100 mM KCl, 2 mM CaCl_2_, 2 mM MgCl_2_, 5 mM DTT, and 0.5% NP40), containing 100 mM PMSF, 10 mM TPCK, and 10 mM TLCK. Elution of the GST fusion protein was performed by resuspension in 3 ml elution buffer (10 mM reduced glutathione in 50 mM Tris/HCl, pH 8.0). After one hour of agitation on a rotary wheel at 4°C, the mixture was resuspended and the supernatant was collected. The elution was repeated four times and the different fractions kept separately. The quantity of the fusion protein recovered was estimated by staining and comparison with a bovine serum albumin calibrated standard after electrophoresis on a denaturing 12% polyacrylamide gel. The protein fractions were lyophilised and kept at -80°C. Before use, the lyophilised fractions were resuspended in PBS, pH 7.5, and dialysed against the same buffer. Control GST protein expressed by the PGEX4T1 vector alone was produced, purified, stored, and used under the same conditions.

### In vitro transcription/translation

The templates for in vitro transcription/translation were prepared as follows: 0.5 μl of each minipreparation DNA plasmid was mixed with 10 μl buffer, 4.8 μl MgCl_2_, 10 μl DMSO, and 62.2 μl water, as described above. The mixture was boiled for five minutes and chilled on ice before the addition of 7.5 μl of water, 2.0 μl of 10 mM dNTPs, and 0.5 μl of Taq polymerase. The preparations were then incubated for 35 cycles at 95°C for one minute, 60°C for one minute, and 72°C for one minute. The in vitro transcription/translation of PCR amplified templates was performed with the TNT lysate reaction mix (Promega, Charbonniéres, France), as recommended by the supplier, with the following modifications: PCR products were extracted once with 200 μl of a 24/1 mixture of chloroform and isoamyl alcohol, and precipitated by the addition of 15 μl 3 M sodium acetate and 500 μl of pre-chilled (-20°C) absolute ethanol. After 10 minutes of incubation in a dry ice/ethanol bath, the DNA precipitate was collected by a 10 minute centrifugation at 12 000 *g *in a microcentrifuge, washed once with 70% chilled ethanol, and dried by speedvac centrifugation at medium temperature. The DNA precipitate was resuspended in 6 μl of sterile distilled water and mixed with 25 μl of the TNT reticulocyte extract, 2 μl of the TNT reaction buffer, 1 μl of T7 RNA polymerase, 1 μl of 1 mM amino acid mixture deprived of methionine, 1 μl of RNAsin ribonuclease inhibitor, and 4 μl of [^35^S] methionine (37 TBq/mmol, 10 mCi/ml). The mixture was incubated for 1.5 hours at 30°C; the in vitro proteins were analysed by electrophoresis on a 15% polyacrylamide gel and visualised by autoradiography.

### Primers

T7AD: 5'-TAA TAC GAC TCA CTA TAG GGA GAC CAC CAC ATG GAT GAT GTA TAT AAC TAT CTA TTC-3'

4ADREV2: 5'-CAG TGA GCG CGC GTA ATA CGA CTC ACT ATA-3'

After synthesis, the oligonucleotides were deprotected by overnight incubation at 55°C, dried in 100 μl fractions with a speedvac with heating, and resuspended in 100 μl of TE (10 mM Tris/HCl, pH 8.0, 1 mM EDTA) before loading on to 1 ml sephadex G25 columns previously prepared in disposable plastic syringes. The flow through contained the purified oligonucleotides.

## Results

The analysis of 93 clones that showed various levels of positivity in the two hybrid screening, identified several new types of potential partners in addition to those that we previously described [7,8]. These potential partners included PAGP2, EF1alpha, SO4A1, Calmyrin, LGALS3BP, IL33, SNRPA and HMGA1.

To check that the candidate clones encoded proteins that could physically interact with CCN3 outside of the yeast nucleus, the plasmids encoding the potential targets were purified after transformation of HB101 bacteria and PCR amplified before being used for in vitro transcription/translation (see materials and methods). The [^35^S] labelled proteins synthesised from the PCR amplified templates were used to run GST pull down assays. Binding of in vitro labelled proteins to GST-CCN3 sepharose beads was revealed after SDS-PAGE analysis of the incubation mixtures.

The variety of proteins detected in this screen fit into the known and postulated biological properties assigned thus far to CCN3. Examination of the literature on these potential partners has revealed new insights into the possible roles of CCN3 in signaling processes associated with inflammation, and has reinforced the idea that nuclear CCN3 variants are likely involved in the regulation of transcription. The properties of these new partners are briefly detailed below.

### Membrane, transport, and recycling events

PGAP2 is a Golgi/ER-resident membrane protein that is involved in the processing of glycosylphosphatidylinositol-anchored proteins (GPI-Aps) that is required for their stable expression at the cell surface [9]. The potential interaction of CCN3 with PGAP2 might provide clues for the post translational processing of CCN3 and its transport through the cell membrane.

The SO4A1 membrane protein is encoded by the SLCO4A1gene [10]. It is a member of the homo sapiens solute carrier organic anion transporter family. Also known as OATP4A1, it mediates the Na(+)-independent transport of organic anions such as thyroid hormones T3 (triiodo-L-thyronine), T4 (thyroxine) and rT3, and of estrone-3-sulfate and taurocholate. The interaction of CCN3 with SO4A1 would be in the same vein as previous observations that reported the CCN3-mediated ion transport [11].

Calmyrin is also known as calcium and integrin binding protein(CIB1) [12]. Interaction of CCN3 with this EF-hand calcium-binding protein [13,14] is in agreement with the participation of CCN3 in calcium signaling and with the physical interaction of CCN3 with integrins. The calmyrin-CCN3 interaction might therefore be part of a multiprotein complex allowing a direct cross talk between regulatory pathways that control the intracellular calcium concentration. Calmyrin is ubiquitously expressed. It is an activating and inhibiting ligand of the inositol 1,4,5 triphosphate receptor (InsPR). Calmyrin was reported to activate InsPR gating in the absence of InsPR [15]. Therefore, our observation raises the possibility that the effects of CCN3 on calcium flux might involve an interactive modulation of the calmyrin-induced gating. Considering that brain is a major site of expression for CCN3 and that calmyrin was detected in difuse and senile plaques, the physical interaction of CCN3 and calmyrin and resulting effects on calcium signaling, might also be of significance in the pathogenesis of Alzheimer's disease.

The EF1 alpha translation elongation factor was identified as a possible partner for several different types of proteins that were assayed in the two hybrid system and interactions with EF1alpha have often been considered as the result of a technical artifact that constitute a drawback of the selective procedure. However, it now well established that EF1alpha is involved in proteasome-directed proteolysis and is essential for ubiquitin-degration of certain proteins [16]. Inasmuch as CCN3 is subject to post-translational proteasome degradation (Bleau et al., submited for publication), the identification of EF1 as one of its potential partners is highly relevant. It is interesting to point out that EF1alpha was recently shown to be a potential oncogene overexpressed in ovarian and breast cancer [17,18] and to play non canonical functions in cytoskeletal remodeling and apoptosis [19].

### Inflammation

LGALS3BP (also known as 90 K, and MAC2 protein) is a 90 Kda secreted protein binds the human macrophage-associated lectin (LGALS3:Lectin galactoside-binding soluble 3) [20,21]. It contains a N-terminal region that shows 45% identity with the extracellular part of the macrophage scavenger receptor (MSR1). The 90 K protein is present in the extracellular matrix of several tissues, as well as in biological fluids. Also known as Mac2-binding protein (M2BP, the former name of galectine 3 is Mac-2) it was also shown to interact with several other extracellular proteins such as collagens, fibronectin, and nidogen [20-23]. In this context, the binding of CCN3 to MAC2 is in line with the interaction of CCN3 with collagen IV. On a physiological standpoint, the interaction of CCN3 and MAC2 is also supported by homotypic cell aggregation induced by galectin binding and integrin-mediated cell adhesion to MAC2. Circulating levels of MAC2 are increased in several cancers. In agreement with a possible connection with CCN3 and inflammation (see below), the production of MAC2 that is trigered by oncogenic transformation and viral infection results in host immune system response via IL2 and induction of other cytokines.

IL 33 is an interleukin-1(IL1)-like cytokine that signals via the IL-1-receptor-related ST2. The ST2 receptor which was an orphan member of the IL-1 family for about 15 years was known as a negative regulator of Toll-like receptor-IL-1 receptor signaling. IL 33 is the specific ligand for ST2 [24,25]. The biological effects of IL33 that are mediated by the ST2 receptor include activation of NFkappaB and MAP kinases, and production of T helper type 2-associated cytokines [26]. Increased ST2 levels in the cerebrospinal fluid (CSF) were associated to the inflammatory reaction in the central nervous system after subarachnoid hemorrhage (SAH) [27]. Therefore, binding of CCN3 to IL33 might participate in the regulation of IL33 biological activity in the context of inflammation.

### Transcriptional events

Nuclear proteins were also identified as potential partners of CCN3. The high mobility group A (HMGA) proteins are non-histone chromosomal proteins implicated in the organization of chromatin structure [28]. They participate in the assembly of multi-protein complexes on the promoter of several inducible genes, and are considered as architectural transcription factors playing key roles in the stereospecificity of transcription. The increased expression of HMGA1 has been associated to both 3T3-L1 adipocytic differentiation, and malignant cells proliferation. Furthermore rearrangements of HMGA genes has been detected in human begnin tumors of mesenchymal origin and has been correlated with highly malignant phenotype in several human tumors [29-34]. Several of these biological properties are shared by CCN3 whose expression was reported to be increased in malignant tumors, to interfere with adipocitic differentiation, and to be involved in the tumorigenic phenotype of CML.

The small nuclear ribonucleoprotein polypeptide A (SNRPA) is one of the three specific proteins found in the U1 snRNP which is the most abundant U snRNP, that are involved in pre-mRNA splicing. It is worth noting that sera from patients with Systemic lupus erythematosus (SLE), and diseases of connective tissue, in which abnormal levels of CCN proteins have been reported, often contain antibodies against sn-RNA associated proteins. SLE is a chronic, remitting, relapsing, inflammatory disorder of connective tissue. It is characterized by involvement of the skin, joints, kidneys, and serosal membranes. An association between chondrodysplasia punctata and maternal SLE was also reported [35].

## Discussion

In this study we identified new potential partners for CCN3 that can be categorized into two main types of proteins: those involved in the metabolic modification and transport of CCN3 and those that point to CCN3 as a factor involved in external and intracellular signaling. Here we highlight the potential roles for CCN3 in inflammatory processes and transcriptional events. In particular, we present data that argue for truncated forms of CCN proteins as mediators of as yet unappreciated nuclear processes.

### Inflammatory processes

Among the new potential partners of CCN3 that were uncovered in this study, IL33 stands as a particularly interesting one. Indeed, the possibility that IL33 signaling functions are modulated through its interaction with CCN3 is in line with previous observations that associated CCN proteins to inflammation. For example, CCN6 (wisp3) was identified as a marker for inflammatory breast cancer [36] and recent studies indicated that expression of ccn6 RNA was considerably increased in Rheumatoid arthritis (RA) and to lesser extent in osteoarthritis (OA) [37]. Stimulation of RA fibroblast-like synoviocytes (FLS) with interleukin 1 or tumor necrosis factor-a, resulted in a significant increase of WISP3 mRNA. These cytokines also increased WISP3 mRNA in OA FLS, but the maximal level in stimulated OA FLS was less than medium-treated RA FLS.

Recent studies revealed induced CCN2 expression in OA, and suggested that CCN2 was involved in inflammatory response and repair process of articular cartilage during arthritis [38]. Both *ccn2 *and *ccn1 *expression was repressed upon Tumor necrosis alpha (TNF-α) stimulation, and both mRNA species were uniformly induced by transforming growth factor (TGF)-β and dexamethasone [39].

In the case of MAV-induced nephroblastomas which represent a unique model of Wilms' tumor [40], we observed that viral infection of tissue targets induced a dramatic inflammation of the cortical region of the kidneys. This inflammation occurred concomitant with an elevated expression of CCN3 in the blastemal cells [Cherel et al. Manuscript in preparation].

On the other hand, CCN3 was induced both at the protein and the RNA level, by TNFalpha and IL1 in melanocytes [M. Fukunaga et al. Submited for publication].

Considering the possibility that CCN3 is interacting with IL33 and is induced by other inflammatory cytokines such as IL1 and the pleiotropic TNF-α, one can predict its involvement in inflammatory responses in tissues if it is detected. For example, a role for CCN3 in OA and RA can be anticipated from its expression in the developing cartilage and bone [[1], Bessette et al. Submited for publication].

In the same vein, the interaction of CCN3 with IL33 combined with the high level of CCN3 expression in the brain, makes it highly probable that CCN3 would be involved in inflammatory responses in the nervous system. We had previously reported that CCN3 expression was detected in astrocytes and that the dorsal root ganglia is a major site for CCN3 expression [41-44]. Myelinated fibers that extend from the cell bodies contained in the dorsal root ganglia constitute the dorsal funiculus (dorsal columns). Upon selective dorsal rhizotomy, a surgical procedure performed to reduce leg muscle stiffness and spasticity in children who have cerebral palsy, CCN3 expression was increased in dorsal funiculus [[45], Unpublished data].

Considering i) that CCN3 is involved in healing and tissue repair, ii) the expression pattern of CCN3 in the nervous system, and iii) the connections of CCN3 with cytokines, makes it a good candidate for playing a role in the inflammatory response associated with neurodegenerative processes. Several animal models are being used to test the potential role of CCN3 in flammatory diseases, sclerosis and neurodegenerescence: the MRL-Lpr/Lpr mice that are considered to have an autoimmune disease similar to human Lupus and the SOD mice that carry a mutated transgene of CuZn superoxide dismutase gene SOD1 which has been associated with amyotrophic lateral sclerosis [46,47].

### Nuclear processes

That CCN3 interacts with proteins involved in the transport and processing of secreted proteins was not surprising. It was neither surprising that CCN3 interacted with receptors at the cell surface and other proteins localized in the extracellular matrix. However, the interaction of CCN3 with rpb7, a subunit of RNA polymerase that is found in the nucleus was unexpected and not fully accepted, until we could demonstrate that the nuclear CCN3 proteins can act as trans-repressor of transcription in cultured cells [48]. However, since our attempts to identify specific DNA binding sites for CCN3 did not allow us to confirm a direct binding of CCN3 on target DNA sequences, we have proposed that CCN3 might act as a co-factor in the multiprotein complexes that govern regulation of transcription.

In support for a role of CCN3 in the regulation of transcription, we had previously reported that the expression of an amino truncated CCN3 protein identified in tumor cells was inducing chicken embryo fibroblast proliferation and transformation. Since our recent results established that CCN3 proteins deprived of signal peptide were routed to the nucleus where they inhibited transcription, one can propose that the oncogenic potential of the amino-truncated CCN3 was resulting from the downregulated expression of antiproliferative or tumor-suppressor genes, among which is CCN3 itself.

The identification of two other nuclear factors as potential partners of CCN3 reinforce the idea that nuclear CCN3 proteins are acting as transcriptional regulators. The functions of these factors strongly suggests that CCN3 might participate in chromatin remodeling that takes place during transcription. In addition to the involvement of HMGA in chromatin structure, rpb7 is believed to participate in the cross talk with regulatory factors during transcription, and SNRPA plays a critical role for the maturation of newly synthesized transcripts.

### Truncated forms of CCN as transcriptional mediators

The physical interaction of CCN3 with several proteins which are part of the transcriptosome might account for the detection of nuclear variants in pathological situations leading to a higher proliferation rate, such as blastemal hyperplasia induced by MAV, or in normal situations such as myoblasts in which high levels of a 32 kDa amino truncated CCN3 was detected. Our previous results [49] indicated that retroviral-induced expression of a CCN3 protein lacking the IGFBP domain was inducing cell proliferation and transformation. We also reported that a 32 kDa CCN3 protein, lacking the two first domains of CCN3, was produced in the supernatant of CCN3-producing cultured cells [50]. It would therefore be of interest to establish whether the production of amino-truncated CCN3 outside of the cell is by any mean related to the detection of truncated CCN3 variants inside the cell.

The lack of reagents allowing a precise caracterisation of the CCN3 proteins detected in immunocytochemistry has hampered the identification of truncated variants in tissues or various origins. The antibodies that were have developed and which specifically recognize the individual structural domains of CCN3 [Lazar et al. Manuscript in preparation] should prove helpful in this task.

Interestingly, the expression of truncated nuclear CCN3 proteins is correlated to the metastatic potential of melanoma tumor cells (Rodolfo M. personal communication). We had previously reported that detection of CCN3 in primary Ewing tumors was associated with a higher risk of developing metastasis [51] It will be interesting to check whether truncated CCN3 proteins were produced in these samples.

New evidence is now accumulating to support the concept that the production of nuclear CCN3 proteins is associated with a higher, or at least disregulated proliferation rate. One key question is to determine whether the production of stable truncated CCN3 protein in the supernatant of cultured cells and in biological fluids such as cerebrospinal fluid and serum, is by any mean related to the detection of nuclear CCN3 variants. It is also worth noting that large amounts of truncated CCN3 proteins are detected in some tissues such as brain [43] and in the cytoplasm of highly proliferative cells [52].

The situation that we described for CCN3 has been reported for other secretory proteins and there are several precedents for secreted cytokines being internalized and routed to the nucleus, either as a whole, or after post transcritional modifications (see review by N. Planque CCS submited). Recent data identified full length CCN3 protein in the nucleus of tumor cells, therefore raising the possibilty that not only truncated forms would be routed to another subcellular compartment, but that in some cases, secretory processes might be altered and lead to the accumulation of CCN3 in the cytoplasm or in the nucleus. Whether the full length CCN3 protein also plays a role in the regulation of transcription remains to be determined. Should this activity be confirmed, the CCN3 protein would definitely acquire the status of « moonlighting protein », as suggested previously [53].

Even though the nuclear localisation of cytokines and other secretory factors has been widely documented over the past decade, the concept is not yet unanimously recognized because it is not in agreeement with dogmas of cellular biology. Most significant progress arose from controversial discoveries such as reverse transcription, splicing, and autocatalytic activty of RNAs in the past. We are confident that nuclear routing of secretory proteins will be fully accepted in the near future and that there will be no doubt that the presence of nuclear CCN3 proteins in the nucleus is not a fantasy, but a true fact.

## References

[B1] Perbal B (2001). NOV (nephroblastoma overexpressed) and the CCN family of genes: structural and functional issues. Mol Pathol.

[B2] Rachfal AW, Brigstock DR (2005). Structural and functional properties of CCN proteins. Vitam Horm.

[B3] Perbal B, Takigawa M (2005). The CCN family of proteins: an overview. Imperial College Press editor The CCN proteins: a new family of cell growth and differentiation regulators.

[B4] Perbal B Communication is the key. Cell Commun Signal.

[B5] Mo FE, Muntean AG, Chen CC, Stolz DB, Watkins SC, Lau LF (2002). CYR61 (CCN1) is essential for placental development and vascular integrity. Mol Cell Biol.

[B6] Ivkovic S, Yoon BS, Popoff SN, Safadi FF, Libuda DE, Stephenson RC, Daluiski A, Lyons KM (2003). Connective tissue growth factor coordinates chondrogenesis and angiogenesis during skeletal development. Development.

[B7] Perbal B (1999). Nuclear localisation of NOVH protein: a potential role for NOV in the regulation of gene expression. Mol Pathol.

[B8] Perbal B, Martinerie C, Sainson R, Werner M, He B, Roizman B The C-terminal domain of the regulatory protein NOVH is sufficient to promote interaction with fibulin 1C: a clue for a role of NOVH in cell-adhesion signaling. Proc Natl Acad Sci USA.

[B9] Tashima Y, Taguchi R, Murata C, Ashida H, Kinoshita T, Maeda Y (2006). PGAP2 is essential for correct processing and stable expression of GPI-anchored proteins. Mol Biol Cell.

[B10] Tamai I, Nezu J, Uchino H, Sai Y, Oku A, Shimane M, Tsuji A (2000). Molecular identification and characterization of novel members of the human organic anion transporter (OATP) family. Biochem Biophys Res Commun.

[B11] Perbal B (2003). The CCN3 (NOV) cell growth regulator: a new tool for molecular medicine. Expert Rev Mol Diagn.

[B12] Naik UP, Patel PM, Parise LV Identification of a novel calcium-binding protein that interacts with the integrin alphaIIb cytoplasmic domain. J Biol Chem.

[B13] Yamniuk AP, Nguyen LT, Hoang TT, Vogel HJ Metal ion binding properties and conformational states of calcium- and integrin-binding protein. Biochemistry.

[B14] Gentry HR, Singer AU, Betts L, Yang C, Ferrara JD, Sondek J, Parise LV Structural and biochemical characterization of CIB1delineates a new family of EF-hand-containing proteins. J Biol Chem.

[B15] White C, Yang J, Monteiro MJ, Foskett JK CIB1, aubiquitously-expressed Ca2+-binding protein-ligand of the InsP3receptor Ca2+-release channel. J Biol Chem.

[B16] Chuang SM, Chen L, Lambertson D, Anand M, Kinzy TG, Madura K (2005). Proteasome-mediated degradation of cotranslationally damaged proteins involves translation elongation factor 1A. Mol Cell Biol.

[B17] Anand N, Murthy S, Amann G, Wernick M, Porter LA, Cukier IH, Collins C, Gray JW, Diebold J, Demetrick DJ, Lee JM (2002). Protein elongation factor EEF1A2 is a putative oncogene in ovarian cancer. Nat Genet.

[B18] Chen L, Madura K Increased proteasome activity, ubiquitin-conjugating enzymes, and eEF1A translation factor detected in breast cancer tissue. Cancer Res.

[B19] Condeelis J (1995). Elongation factor 1 alpha, translation and the cytoskeleton. Trends Biochem Sci.

[B20] Sasaki T, Brakebusch C, Engel J, Timpl R Mac-2 binding protein is a cell-adhesive protein of the extracellular matrix which self-assembles into ring-like structures and binds beta1 integrins, collagens and fibronectin. EMBO J.

[B21] Koths K, Taylor E, Halenbeck R, Casipit C, Wang A Cloning and characterization of a human Mac-2-binding protein, a new member of the superfamily defined by the macrophage scavenger receptor cysteine-rich domain. J Biol Chem.

[B22] Rosenberg I, Cherayil BJ, Isselbacher KJ, Pillai S Mac-2-binding glycoproteins. Putative ligands for a cytosolicbeta-galactoside lectin. J Biol Chem.

[B23] Inohara H, Raz A Identification of human melanoma cellular and secreted ligands for galectin-3. Biochem Biophys Res Commun.

[B24] Dinarello CA (2005). An IL-1 family member requires caspase-1processing and signals through the ST2 receptor. Immunity.

[B25] Schmitz J, Owyang A, Oldham E, Song Y, Murphy E, McClanahan TK, Zurawski G, Moshrefi M, Qin J, Li X, Gorman DM, Bazan JF, Kastelein RA (2005). IL-33, an interleukin-1-like cytokine that signals via the IL-1 receptor-related protein ST2 and induces T helper type 2-associated cytokines. Immunity.

[B26] Takezako N, Hayakawa M, Hayakawa H, Aoki S, Yanagisawa K, Endo H, Tominaga S ST2 suppresses IL-6 production via the inhibition of IkappaB degradation induced by the LPS signal in THP-1 cells. Biochem Biophys Res Commun.

[B27] Kanda M, Ohto-Ozaki H, Kuroiwa K, Tominaga S, Watanabe E, Iwahana H (2006). Elevation of ST2 protein levels in cerebrospinal fluid following subarachnoid hemorrhage. Acta Neurol Scand.

[B28] Lovell-Badge R Developmental genetics. Living with bad architecture. Nature.

[B29] Melillo RM, Pierantoni GM, Scala S, Battista S, Fedele M, Stella A, De Biasio MC, Chiappetta G, Fidanza V, Condorelli G, Santoro M, Croce CM, Viglietto G, Fusco A (2001). Critical role of the HMGI(Y) proteinsin adipocytic cell growth and differentiation. Mol Cell Biol.

[B30] Abe N, Watanabe T, Izumisato Y, Suzuki Y, Masaki T, Mori T, Sugiyama M, Fusco A, Atomi Y (2003). High mobility group A1 is expressed in metastatic adenocarcinoma to the liver and intrahepaticcholangio carcinoma, but not in hepatocellular carcinoma: its potential use in the diagnosis of liver neoplasms. J Gastroenterol.

[B31] Masciullo V, Baldassarre G, Pentimalli F, Berlingieri MT, Boccia A, Chiappetta G, Palazzo J, Manfioletti G, Giancotti V, Viglietto G, Scambia G, Fusco A (2003). HMGA1 protein over-expression is a frequent feature of epithelial ovarian carcinomas. Carcinogenesis.

[B32] Pierantoni GM, Agosti V, Fedele M, Bond H, Caliendo I, Chiappetta G, Lo Coco F, Pane F, Turco MC, Morrone G, Venuta S, Fusco A High-mobility group A1 proteins are overexpressed in human leukaemias. Biochem J.

[B33] Ashar HR, Fejzo MS, Tkachenko A, Zhou X, Fletcher JA, Weremowicz S, Morton CC, Chada K Disruption of the architectural factor HMGI-C: DNA-binding AT hook motifs fused in lipomas to distinct transcriptional regulatory domains. Cell.

[B34] Schoenmakers EF, Wanschura S, Mols R, Bullerdiek J, Van den Berghe H, Van de Ven WJ (1995). Recurrent rearrangements in the highmobility group protein gene, HMGI-C, in benign mesenchymal tumours. Nat Genet.

[B35] Kozlowski K, Basel D, Beighton P (2004). Chondrodysplasiapunctata in siblings and maternal lupus erythematosus. Clin Genet.

[B36] Kleer CG, Zhang Y, Pan Q, van Golen KL, Wu ZF, Livant D, Merajver SD WISP3 is a novel tumor suppressor gene of inflammatory breast cancer. Oncogene.

[B37] Cheon H, Boyle DL, Firestein GS (2004). Wnt1 inducible signaling pathway protein-3 regulation and microsatellite structure in arthritis. J Rheumatol.

[B38] Omoto S, Nishida K, Yamaai Y, Shibahara M, Nishida T, Doi T, Asahara H, Nakanishi T, Inoue H, Takigawa M (2004). Expression and localization of connective tissue growth factor (CTGF/Hcs24/CCN2) in osteoarthritic cartilage. Osteoarthritis Cartilage.

[B39] Moritani NH, Kubota S, Sugahara T, Takigawa M Comparable response of ccn1 with ccn2 genes upon arthritis: An in vitro evaluation with a human chondrocytic cell line stimulated by a set ofcytokines. Cell Commun Signal.

[B40] Perbal B (1994). Contribution of MAV-1-induced nephroblastoma tothe study of genes involved in human Wilms' tumor development. Crit Reev Oncog.

[B41] Su BY, Cai WQ, Zhang CG, Su HC, Perbal B (1998). A developmental study of novH gene expression in human central nervous system. C R Acad Sci III.

[B42] Su BY, Cai WQ, Xiong Y, Zhang CG, Perbal B (2000). [Relationships between learning and memory and expression of nov gene of rats]. Sheng Li Xue Bao.

[B43] Su BY, Cai WQ, Zhang CG, Martinez V, Lombet A, Perbal B (2001). The expression of ccn3 (nov) RNA and protein in the rat central nervous system is developmentally regulated. Mol Pathol.

[B44] Kocialkowski S, Yeger H, Kingdom J, Perbal B, Schofield PN (2001). Expression of the human NOV gene in first trimester fetal tissues. Anat Embryol (Berl).

[B45] Perbal B, Brigstock DR, Lau LF (2003). Report on the second international workshop on the CCN family of genes. Mol Pathol.

[B46] Tada Y, Nagasawa K, Ho A, Morito F, Koarada S, Ushiyama O, Suzuki N, Ohta A, Mak TW Role of the costimulatory molecule CD28 in the development of lupus in MRL/lpr mice. J Immunol.

[B47] Shibata N (2001). Transgenic mouse model for familial amyotrophic lateral sclerosis with superoxide dismutase-1 mutation. Neuropathology.

[B48] Planque N, Long Li C, Saule S, Bleau AM, Perbal B Nuclear addressing provides a clue for the transforming activity of amino-truncated CCN3 proteins. J Cell Biochem.

[B49] Joliot V, Martinerie C, Dambrine G, Plassiart G, Brisac M, Crochet J, Perbal B (1992). Proviral rearrangements and overexpression of a new cellular gene (nov) in myeloblastosis-associated virus type 1-induced nephroblastomas. Mol Cell Biol.

[B50] Chevalier G, Yeger H, Martinerie C, Laurent M, Alami J, Schofield PN, Perbal B (1998). novH: differential expression in developing kidney and Wilm's tumors. Am J Pathol.

[B51] Manara MC, Perbal B, Benini S, Strammiello R, Cerisano V, Perdichizzi S, Serra M, Astolfi A, Bertoni F, Alami J, Yeger H, Picci P, Scotlandi K (2002). The expression of ccn3(nov) gene in musculoskeletal tumors. Am J Pathol.

[B52] Kyurkchiev S, Yeger H, Bleau AM, Perbal B Potential cellular conformations of the CCN3(NOV) protein. Cell Commun Signal.

[B53] Perbal B (2005). Moonlight in Saint-Malo. Report and abstracts of the 3rd International Workshop on the CCN Family of Genes. StMalo, France, 20–23 October 2004. J Clin Pathol.

